# Genotyping of *Toxoplasma gondii* Isolates from Wild Boars in Peninsular Malaysia

**DOI:** 10.1371/journal.pone.0061730

**Published:** 2013-04-17

**Authors:** Vignesh R. Puvanesuaran, Rahmah Noordin, Venugopal Balakrishnan

**Affiliations:** Institute for Research in Molecular Medicine (INFORMM), Universiti Sains Malaysia, Penang, Malaysia; University of Oklahoma Health Sciences Center, United States of America

## Abstract

*Toxoplasma gondii* is a parasitic protozoan that infects nearly one-third of the world population. The present study was done to isolate and genotype *T. gondii* from wild boar from forests of Pahang, Malaysia. A total of 30 wild boars' blood, heads and hearts were obtained for this study and 30 (100.0%) were found to be seropositive when assayed with modified agglutination test (MAT≥6). The positive samples were inoculated into mice and *T. gondii* was only isolated from samples that had strong seropositivity (MAT≥1∶24).The isolates were subjected to PCR-RFLP analysis and all the Peninsular Malaysia isolates of *T. gondii* are of clonal type I.

## Introduction

*Toxoplasma gondii* is a cosmopolitan protozoon that causes an important zoonosis. It is considered as the most successful parasitic pathogen worldwide [1]. Although all warm blooded animals including marine mammals [2] and birds are susceptible to the infection, only felids are the definitive host of the parasite. Infected felids shed environmentally resistant oocysts which become infectious upon sporulation [3]. Once these oocysts are ingested, the protozoa alters its form to rapidly dividing tachyzoite, and then to slower dividing bradyzoites that encysts in brain, muscle and nervous tissues [4]. This provides the parasite an important pathway to successfully transmit the infection via consumption of uncooked or undercooked meat [5]. One such incidence was reported by Choi et al. [6] who described a case whereby *T. gondii* infection was acquired from eating raw wild boar (*Sus scrofa*) meat that led to blindness and loss of vision.

Wild boar meat is considered a delicacy in Malaysia and often enjoyed by the ethnic Chinese community. The Act 76 of Malaysian Protection of Wild Life Act (1972) lists wild boar under “Protected Wild Animals” category. However, the act also recognizes wild boar as a game animal and thus hunting is allowed but the numbers that can be hunted are seriously limited and the licensed hunters are monitored accordingly.

Genotyping study on local isolates of *T. gondii* in South-East Asian region is minimal with only one report from Vietnam by Dubey et al. [7]. In relation to this, only seroprevalence of *T. gondii* in animals has been studied in Malaysia. In one such study, Rajamanickam et al. [8] reported 15.6% infection in the pigs; while in another study, Chandrawathani et al. [9] reported no infection in pigs. With the aim of increasing our knowledge on the *T. gondii* genotypes in this region, the present study was designed to determine whether the local wild boars in Malaysia are infected with *T. gondii*.

## Materials and Methods

### Wild boar sampling

All the animals used in this study originated from forests in Pahang, a state in the east coast of Peninsular Malaysia. A total of 30 samples from adult boars were obtained from multiple licensed hunters who hunted the wild boars with rifles. The hunters did not hunt the animals especially for the purpose of this study; rather, they hunt the boars for the meat and the samples collected for this study are usually deemed as waste by them. Blood, brain and heart of the animals were collected and shipped to our laboratory in ice boxes to maintain the temperature at 4°C. The samples were processed within 5 days from the day it was collected from the hunters. Serum samples were kept at −80°C.

### Serological examination

Serum samples from the wild boars were tested for IgG antibodies using the modified agglutination test (MAT), as described by Dubey and Desmonts [10]. Whole RH tachyzoites were obtained from intraperitoneum of experimentally infected mice and stored in −20°C until further use. The samples were diluted two fold starting at 1∶6 dilutions, and both positive and negative controls were included. Agglutination reaction at a titer of 1∶6 and above was regarded as positive.

### Animal ethics

Approval (USM/PPSF/50(088)Jld.2) was obtained from USM animal research ethics committee for use of mice for intraperitoneal infection of wild boar samples and to maintain the RH strain *in-vivo*. All animal work carried out in this research were in accordance to the guidelines set by The Animal Research Ethics Committee of USM (JEHUSM).

### Isolation and bioassay for *T. gondii* infection

The brain and heart from the same wild boar sample with MAT titer of ≥1∶6 were pooled, homogenized with phosphate buffered saline (PBS), digested with acidic pepsin solution by incubation at 37°C for 1 hour, and then neutralized with 1.2% sodium bicarbonate as described by Dubey [11]. The neutralized mixture was then filtered through a 100 µm glass filter and the filtrate was pelleted in order to remove unwanted particles and concentrate the parasite in order to reduce the number of mice needed for infection as explained by Puvanesuaran et al. [12]. The pellet was then mixed with PBS containing 100 units of penicillin and 100 µg of streptomycin per ml before infecting a group of two mice per sample. Mice from Swiss albino strain used in this study were obtained from USM Animal facility. Brains of the mice that died were collected and stored in PBS at 4°C. In addition, intraperitoneal fluids were collected if present. Mice that survived 21 days post-infection (p.i) were bled through their orbital sinus as described by Hoff [13] to check for *T. gondii* antibodies by MAT. Surviving mice were euthanized at 45 days p.i and their brains were collected and stored in PBS at 4°C.

The mice organs were separately homogenized and centrifuged at 500× *g* for 2 min. The resulting supernatant was collected and pelleted by centrifugation at 3,000× *g* for 15 min. The pellet was use for DNA extraction using QIAamp DNA Mini Kit (Qiagen, Hilden, Germany), according to the protocol suggested by the manufacturer. The quality and concentration of the isolated DNA was checked using the Nanophotometer (Implen, Munich, Germany), then stored at −20°C. The DNA templates used in this study were between 50–100 ng/µl and the ratios of A_260_/A_280_ were between 1.8–2.0.

### PCR-RFLP analysis

PCR amplification was performed using eight different markers (*3′ SAG2*, *5′ SAG2*, *BTUB*, *cB21-4*, *GRA1*, *GRA6*, *SAG3* and *SRS1*) under previously optimized conditions as reported by Puvanesuaran et al. [14]. The PCR amplification process was carried out in the 96-well thermal cycler (MyCycler, Bio-Rad, California, USA). The reaction mixture was denatured at 95°C for 5 minutes; followed by denaturing step for 30 seconds, annealing for 30 seconds and extension at 72°C for 1 minute for 40 cycles. Final extension was at 72°C for 10 minutes. The PCR products were purified using QIAquick PCR Purification Kit (Qiagen, Hilden, Germany) and digested using appropriate restriction enzymes as listed in Table 1; each digestion was carried out according to the conditions suggested by the manufacturer (New England Biolabs, Massachusetts, USA). The RFLP analysis was carried out and analyzed by referring to the data in *Toxoplasma* Genome Map Database **[** http://toxomap.wustl.edu/Toxo_Genetic_Map_Table.html **]**. The positive controls used in this study were from previously isolated genomic DNA from RH (type I), ME49 (type II) and VEG (type III) strains of *T. gondii*. Water was used as negative control in this study.

**Table 1:** RFLP markers and restriction enzymes used in this study.

Markers	Restriction Enzyme
***3′-SAG2***	*Hha* I
***5′-SAG2***	*Sau3A* I
***BTUB***	*BsiE* I and *Taq* I
***cB21-4***	*Hae* III
***GRA1***	*Hind* III and *Nla* IV
***GRA6***	*Mse* I
***SAG3***	*Nci* I
***SRS1***	*ApaL* I and *Hae* II

## Results and Discussion

The peninsular of Malaysia has an abundance of rainforest and is estimated to contain 20% of all animal species in the world [15]. The vast forests in the state of Pahang are a known domain for a variety of wild felids such as tigers (*Panthera tigris*), panthers (*Panthera pardu*s), clouded leopards (*Neofelis nebulosa*) and many others. Sampling of wild boar samples from this area can help determine if the soil is contaminated with *T. gondii* and of which genotype.

Shaapan et al. [16] reported that MAT was more specific and sensitive in comparison to ELISA, thus MAT was used as the serological test in this study. All the 30 samples were found to have a minimal MAT titer of 1∶6 and were bioassayed into 30 groups of mice. Six samples (6/30, 20.0%) had MAT titer of ≥1∶24 which indicated strong positive reaction [17]. The results of MAT are summarized in Table 2. Among the mice that were inoculated with samples from animals that had MAT titer of ≥1∶24, 11 (11/12, 91.67%) died within 1 week p.i. Estimated time of death in hours p.i for these mice are listed in Table 2, while the symptoms observed on these mice were consistent to the observations made by Hisaeda et al. [18] on outbred mice infected with RH (type I) strain of *T. gondii*. This is also an indicator to the virulence of the isolates which are similar to the archetypal type I strains that are uniformly lethal in outbred mice [19]. These mice showed signs of sickness as described by Hrda et al. [20]; MAT was not performed on these mice as blood was not collected from the carcasses while the organs were harvested. The rest of the infected mice were tested for *T. gondii* serology; the results showed that 49 (49/49, 100.0%) had MAT titer of ≤1∶24. These results too have been summarized in Table 2 along with information on the number of successful isolations obtained from the infected mice.

**Table 2:** MAT titer of wild boar samples, isolation information from infected mice and MAT titer results of infected mice.

Sample ID	MAT titer of wild boar	Isolation in mice	MAT titer of mice	Time of death (p.i)
WB 1	96	2/2	ND^a^,ND	72 h/72 h
WB 2	64	2/2	ND,ND	96 h/96 h
WB 3	6	0/2	0,0	E/E
WB 4	6	0/2	0,0	E/E
WB 5	6	0/2	0,6	E/E
WB 6	6	0/2	0,0	E/E
WB 7	6	0/2	0,12	E/E
WB 8	6	0/2	0,0	E/E
WB 9	24	2/2	ND,ND	144 h/144 h
WB 10	24	1/2	ND,6	144 h/144 h
WB 11	6	0/2	0,0	E/E
WB 12	6	0/2	0,0	E/E
WB 13	6	0/2	6,6	E/E
WB 14	6	0/2	0,0	E/E
WB 15	64	2/2	ND,ND	96 h/120 h
WB 16	48	2/2	ND,ND	120 h/120 h
WB 17	6	0/2	12,0	E/E
WB 18	6	0/2	0,0	E/E
WB 19	6	0/2	0,0	E/E
WB 20	6	0/2	0,0	E/E
WB 21	12	0/2	6,12	E/E
WB 22	6	0/2	0,6	E/E
WB 23	12	0/2	12,6	E/E
WB 24	6	0/2	0,0	E/E
WB 25	6	0/2	0,0	E/E
WB 26	6	0/2	0,0	E/E
WB 27	6	0/2	0,0	E/E
WB 28	6	0/2	6,0	E/E
WB 29	6	0/2	0,0	E/E
WB 30	6	0/2	6,0	E/E

**ND^a^: No data.**

**E: Euthanized.**

**h: hours.**

Dubey [17] reported that when MAT titers are 1∶24, the specificity for detection of *T. gondii* antibodies are very high. This study showed that 6 of the 30 (20.0%) wild boar samples had MAT titer of ≥1∶24. The wild boar samples with lower titers were still processed and inoculated into mice as there have been studies reporting isolation of viable *T. gondii* from samples of low MAT titers (MAT>1∶5) [21]. Recent serological studies carried out in China by Cong et al. [22] to detect seropositivity of *T. gondii* in market sold animals also used similar MAT titer. However, when inoculated into mice only the samples with MAT titer of ≥1∶24 had *T. gondii* infection in current study, thus corroborating the above observation by Dubey [17]. Nevertheless, not all samples with MAT titer of ≥1∶24 had successful isolation of the parasite from the infected mice as observed in sample WB10 in which only one mouse had the infection resulting in a successful isolate, while the other mouse had a MAT titer of 1∶6 and was negative by PCR amplification. Thus for future studies, MAT titer should be set at higher values (≥1∶24) when the parasite must be isolated to reduce false positive results as well as to help reduce the number of mice sacrificed for similar studies.

The 49 mice which survived 45 p.i were euthanized and DNA was extracted from their brains, and tested with two PCR markers (*GRA6* and *cB21-4*), and the results were negative for all samples. DNA extracted from mice that died within a week p.i was also tested using the same markers and all were positive. The positive samples were then subjected to PCR amplification and RFLP analysis using other markers namely *3′SAG2*, *5′SAG2*, *BTUB*, *GRA1*, *SAG3* and *SRS1*. The results are summarized in Table 3.

**Table 3:** Genotypes of *T. gondii* isolates from wild boar in Pahang, Malaysia.

Isolate Designation	*3′+5′ SAG2*	*BTUB*	*cB21-4*	*GRA1*	*GRA6*	*SAG3*	*SRS1*	Genotype
WB1-a*	I	I	I	I	I	I	I	I
WB1-b*	I	I	I	I	I	I	I	I
WB2-a	I	I	I	I	I	I	I	I
WB2-b	I	I	I	I	I	I	I	I
WB9-a	I	I	I	I	I	I	I	I
WB9-b	I	I	I	I	I	I	I	I
WB10-a	I	I	I	I	I	I	I	I
WB15-a	I	I	I	I	I	I	I	I
WB15-b	I	I	I	I	I	I	I	I
WB16-a	I	I	I	I	I	I	I	I
WB16-b	I	I	I	I	I	I	I	I
RH	I	I	I	I	I	I	I	Ref# (I)
ME49	II	II	II	II	II	II	II	Ref (II)
VEG	III	III	III	III	III	III	III	Ref (III)

* **a,b: are isolates from two different mice.**

# **: Indicates reference strains.**

All the isolates obtained in this study were determined to be of type I strain of *T. gondii*. Type I strains are known to be very virulent to mice and causes parasitemia; they are also closely associated with human congenital toxoplasmosis [23]. The only other similar study carried out in this region was on dogs in Vietnam; it was observed that among the 42 samples tested, 50% were seropositive and two different atypical strains were successfully isolated from 8 of the 21 positive samples [7].

To the best of our knowledge, this is the first reported study on *T. gondii* serology and genotyping from wild boar samples in this region. Nimir et al. [24] reported that in Malaysia, most people who were found seropositive to *T. gondii* infection were from rural areas and at some point of their life has consumed undercooked or raw meat. Since free-range meat products are quite commonly consumed in Malaysia and regional countries, further genotyping of local isolates of *T. gondii* should be carried out.

## References

[1] Katzer F, Brulisauer F, Collantes-Fernandez E, Bartley PM, Burrells A, et al (2011) Increased *Toxoplasma gondii* positivity relative to age in 125 Scottish sheep flocks; evidence of frequent acquired infection. Vet Res 42: 121. 22189159 10.1186/1297-9716-42-121 PMC3284422

[2] Mazzariol S, Marcer F, Mignone W, Serracca L, Goria M, et al (2012) *Dolphin Morbillivirus* and *Toxoplasma gondii* coinfection in a Mediterranean fin whale (*Balaenoptera physalus*). BMC Vet Res 8: 20. 22397492 10.1186/1746-6148-8-20 PMC3319419

[3] Herrmann DC, Pantchev N, Vrhovec MG, Barutzki D, Wilking H, et al (2010) Atypical *Toxoplasma gondii* genotypes identified in oocysts shed by cats in Germany. Int J Parasitol 40: 285–292. 19695254 10.1016/j.ijpara.2009.08.001

[4] Tomavo S (2001) The differential expression of multiple isoenzyme forms during stage conversion of *Toxoplasma gondii*: an adaptive developmental strategy. Int J Parasitol 31: 1023–1031. 11429165 10.1016/s0020-7519(01)00193-x

[5] Bokken GC, Bergwerff AA, van Knapen F (2012) A novel bead-based assay to detect specific antibody responses against *Toxoplasma gondii* and *Trichinella spiralis* simultaneously in sera of experimentally infected swine. BMC Vet Res 8: 36. 22453053 10.1186/1746-6148-8-36 PMC3348074

[6] Choi WY, Nam HW, Kwak NH, Huh W, Kim YR, et al (1997) Foodborne outbreaks of human toxoplasmosis. J Infect Dis 175: 1280–1282. 9129105 10.1086/593702

[7] Dubey JP, Huong LT, Sundar N, Su C (2007) Genetic characterization of *Toxoplasma gondii* isolates in dogs from Vietnam suggests their South American origin. Vet Parasitol 146: 347–351. 17442492 10.1016/j.vetpar.2007.03.008

[8] Rajamanickam C, Cheah TS, Paramasvaran S (1990) Antibodies to *Toxoplasma gondii* from domestic animals in Malaysia. Trop Anim Health Prod 22: 61–62. 2321262 10.1007/BF02243502

[9] Chandrawathani P, Nurulaini R, Zanin C, Premaalatha B, Adnan M, et al (2008) Research Note Seroprevalence of *Toxoplasma gondii* antibodies in pigs, goats, cattle, dogs and cats in peninsular Malaysia. Trop Biomed 25: 257–258. 19287367

[10] Dubey JP, Desmonts G (1987) Serological responses of equids fed *Toxoplasma gondii* oocysts. Equine Vet J 19: 337–339. 3622463 10.1111/j.2042-3306.1987.tb01426.x

[11] Dubey JP (1998) Refinement of pepsin digestion method for isolation of *Toxoplasma gondii* from infected tissues. Vet Parasitol 74: 75–77. 9493311 10.1016/s0304-4017(97)00135-0

[12] Puvanesuaran VR, Ibrahim N, Noordin R, Balakrishnan V (2012) Isolation of viable *Toxoplasma gondii* cysts from brain samples for oral infection. Eur Rev Med Pharmacol Sci 16: 1179–1183. 23047500

[13] Hoff J (2000) Methods of blood collection in the mouse. Lab Anim 29: 47–53.

[14] Puvanesuaran VR, Ibrahim N, Noordin R, Balakrishnan V (2012) Variations in annealing temperature and Magnesium chloride concentration for eight RFLP markers of *Toxoplasma gondii* . Int J Pharm Pharm Sci 4: 220–224.

[15] Alexander J (2006) Malaysia Brunei & Singapore: Cadogan Guides.

[16] Shaapan RM, El-Nawawi FA, Tawfik MA (2008) Sensitivity and specificity of various serological tests for the detection of *Toxoplasma gondii* infection in naturally infected sheep. Vet Parasitol 153: 359–362. 18406534 10.1016/j.vetpar.2008.02.016

[17] Dubey JP (1997) Validation of the specificity of the modified agglutination test for toxoplasmosis in pigs. Vet Parasitol 71: 307–310. 9299699 10.1016/s0304-4017(97)00016-2

[18] Hisaeda H, Sakai T, Ishikawa H, Maekawa Y, Yasutomo K, et al (1997) Heat shock protein 65 induced by gammadelta T cells prevents apoptosis of macrophages and contributes to host defense in mice infected with *Toxoplasma gondii* . J Immunol 159: 2375–2381. 9278328

[19] Su C, Howe DK, Dubey JP, Ajioka JW, Sibley LD (2002) Identification of quantitative trait loci controlling acute virulence in Toxoplasma gondii. Proc Natl Acad Sci U S A 99: 10753–10758. 12149482 10.1073/pnas.172117099 PMC125035

[20] Hrda S, Votypka J, Kodym P, Flegr J (2000) Transient nature of *Toxoplasma gondii*-induced behavioral changes in mice. J Parasitol 86: 657–663. 10958436 10.1645/0022-3395(2000)086[0657:TNOTGI]2.0.CO;2

[21] Sreekumar C, Graham DH, Dahl E, Lehmann T, Raman M, et al (2003) Genotyping of *Toxoplasma gondii* isolates from chickens from India. Vet Parasitol 118: 187–194. 14729166 10.1016/j.vetpar.2003.10.018

[22] Cong W, Huang SY, Zhou DH, Xu MJ, Wu SM, et al (2012) First report of *Toxoplasma gondii* infection in market-sold adult chickens, ducks and pigeons in northwest China. Parasit Vectors 5: 110. 22676311 10.1186/1756-3305-5-110 PMC3414767

[23] Howe DK, Sibley LD (1995) *Toxoplasma gondii* comprises three clonal lineages: correlation of parasite genotype with human disease. J Infect Dis 172: 1561–1566. 7594717 10.1093/infdis/172.6.1561

[24] Nimir A, Othman A, Ee S, Musa Z, Majid IA, et al (2010) Latent toxoplasmosis in patients with different malignancy: a hospital based study. J Clin Med Res 2: 117–120. 21629523 10.4021/jocmr2010.06.375w PMC3104645

